# Phosphatase PP2A promotes RTA dephosphorylation to impair KSHV lytic replication

**DOI:** 10.1371/journal.ppat.1013731

**Published:** 2025-12-03

**Authors:** Lei Bai, Lianghui Dong, Jiazhen Dong, Xiaowei Liang, Jiangwei Peng, Yuncai Chen, Xiaoyi Sun, Yuting Chen, Xintong Li, Hua Cai, Jing Huang, Zixu Cao, Ke Lan

**Affiliations:** 1 State Key Laboratory of Virology and Biosafety, College of Life Sciences, Wuhan University, Wuhan, China; 2 Frontier Science Center for Immunology and Metabolism, Wuhan University, Wuhan, China; 3 TaiKang Center for Life and Medical Sciences, Wuhan University, Wuhan, China; Tulane University School of Medicine, UNITED STATES OF AMERICA

## Abstract

Kaposi’s Sarcoma-associated herpesvirus (KSHV) is a human γ herpesvirus that establishes two different phases in its life cycle, the latency and lytic replication. KSHV-encoded replication and transcription activator (RTA), an immediate-early master switch protein, plays a central role in switching the viral latency to lytic replication. Extensive studies have described the mechanisms that RTA functions as a transcription factor to activate its downstream viral genes expression, initiating lytic replication. Phosphorylation of RTA has been shown to be critical for its function, but the regulatory mechanisms of phosphorylation and dephosphorylation of RTA have not been fully elucidated. In this study, we showed that RTA interacts with the scaffold protein PPP2R1A of phosphatase PP2A. We next demonstrated that PPP2R1A overexpression and a pharmacological agonist of PP2A, forskolin, both inhibit viral genes expression and impair the production of KSHV progeny virions during viral lytic replication. The underlying mechanism involves RTA dephosphorylation mediated by phosphatase PP2A, which impaired the transcription activity of RTA and therefore suppressed KSHV lytic replication. Interestingly, to evade this host antiviral mechanism, KSHV RTA can promote PPP2R1A degradation through ubiquitin-proteasome pathway. Taken together, we identified that the scaffold protein PPP2R1A is a new binding partner of RTA, and the interaction induces RTA dephosphorylation mediated by phosphatase PP2A, impairing KSHV lytic replication, which provide new insights into the development of novel antiviral strategies.

## Author summary

The phosphorylation and dephosphorylation of RTA play critical roles in RTA expression and function, but the underlying mechanisms of this process have not been fully elucidated. Here, we identified PPP2R1A, a subunit and scaffold protein of phosphatase PP2A, as a new binding protein of RTA. PP2A is involved in several important biological processes, but the roles in KSHV lytic replication remains unclear. Our findings demonstrated that PP2A promotes RTA dephosphorylation, which impairs the transcription activity of RTA, therefore suppresses KSHV lytic replication. Moreover, KSHV RTA can promote PPP2R1A degradation through ubiquitin-proteasome pathway during lytic replication. These findings revealed new mechanisms of RTA dephosphorylation, broaden our understanding about KSHV lytic replication and identified phosphatase PP2A might be a promising therapeutic target for KSHV related diseases.

## Introduction

Kaposi’s sarcoma-associated herpesvirus (KSHV), also known as human herpesvirus 8 (HHV8), is a double-stranded DNA virus which belongs to the subfamily of *Gammaherpesvirinae*. KSHV was first identified in AIDS-associated Kaposi’s sarcoma (AIDS-KS) tissues, and has since been strongly associated with several human diseases, including endothelial-derived Kaposi’s sarcoma (KS), a B cell malignancy named as primary effusion lymphoma (PEL), a subset of multicentric Castleman’s disease (MCD), and KSHV-associated inflammatory cytokine syndrome (KICS) [1–4]. Similar to other herpesvirus, KSHV establishes two phases of its life cycle, latency and lytic replication. During the latent phase, the viral genome forms covalently closed circular episome tethered to the host chromosome and only limited latent genes are expressed without viral progeny produced [5–7]. In contrast, lytic replication induces extensive viral gene expression, produces infectious virions and leads to the death of the host cells [8]. Under specific physiological conditions, KSHV in latently infected cells can be induced to enter the lytic cycle, producing viral progeny that can infect new target cells [9,10].

Two virus-encoded genes LANA (latency-associated nuclear antigen) and RTA (replication and transcription activator) play a critical role in the regulation of viral latency and lytic replication [11–19]. RTA, encoded by ORF50 of KSHV, is an immediate early protein, working as a key master switch for viral lytic cycle. It has been reported that being a viral transcriptional factor, RTA alone is sufficient to drive the latently infected cells to undergo the entire viral lytic replication cycle by trans-activating the expression of other downstream lytic immediate-early genes, including K8, vIL-6 and PAN [12,15,20,21]. RTA has 691 amino acids, and its theoretical molecular weight is about 74 kDa. However, in immunoblot analysis, RTA protein migrates with an apparent molecular mass of about 110 kDa, suggesting RTA exists extensive post-translational modifications, including phosphorylation and SUMOylating, which have a critical impact on RTA expression and function [22,23]. For instance, the phosphorylation of the Ser-634 and Ser-636 of RTA can enhance its transcriptional regulation in lytic reactivation cycle [23]. However, the regulatory mechanisms of RTA dephosphorylation have not been fully elucidated.

Phosphatases (PSP) play critical roles in catalyzing the dephosphorylation of the serine (Ser) or threonine (Thr) proteins, and participate in nearly every biological events in eukaryotic cells, including signal transduction pathways, the regulation of cell cycle, stress responses and defense, etc [24]. PSPs comprise of three major families: phosphoprotein phosphatases (PPPs), metal-dependent phosphatases (PPMs) and aspartate-dependent phosphatases. PPP family are signal transducing enzymes that dephosphorylate cellular phosphoproteins, which has been divided into seven subfamilies, PP1, PP2A, PP2B, PP4, PP5, PP6 and PP7, harboring distinct structures and enzymatic mechanisms [25–27]. The holoenzyme of PPP family consists of one or more regulatory subunits, which target substrates, modulate enzymatic activity, and regulate subcellular localization, allowing for precise and dynamic control of protein phosphorylation events and eventually influencing various cellular processes [24,28]. Protein phosphatase type 2A (PP2A), belonging to the PPP family, consists of three different subunits: the structural scaffold A subunit, the catalytic C subunit and regulatory B subunits. Of note, A and C subunits have two isoforms, α and β, of which the α isoform is the most commonly expressed in most human cell types, while B subunit has at least 21 different members categorized into four different subfamilies [29,30]. Besides, each of these B subunits possesses two to five isoforms and several splice variants with almost no sequence similarities, which determine the immense diversity and are responsible for substrate specificity and spatial localization of PP2A holoenzymes [31–34]. Due to the huge structural diversity, PP2A works as various components to play roles in important signaling pathways, including PI3K, mTOR, MAPK pathways, etc [35,36]. Additionally, PP2A also interacts with the oncoprotein cMyc, apoptosis proteins Bcl2, cell cycle regulator pRb, suggesting PP2A plays a critical role in several biological processes [37,38]. However, the function and mechanism of PP2A in KSHV life cycle remain elusive, encouraging further investigation.

Based on our previous mass spectrometry analysis of KSHV RTA, PPP2R1A, a scaffold protein belonging to the structural scaffold A subunit of PP2A holoenzymes, was the potential interacting partner of RTA [39]. In this study, we firstly verified the interaction between RTA and PPP2R1A. We then demonstrated that PPP2R1A overexpression and a pharmacological agonist of PP2A, Forsklin, both inhibit the expression of lytic genes and impair the production of viral progenies. We further determined that PP2A has the capacity to promote the dephosphorylation of RTA, and therefore suppresses the transactivation mediated by RTA during lytic replication. Moreover, we demonstrated that RTA promotes PPP2R1A degradation with a proteasome pathway during lytic replication to counteract the antiviral activity of PP2A. Taken together, these results reveal that the scaffold protein PPP2R1A of phosphatase PP2A is a new host binding partner of RTA that impairs viral lytic replication through promoting RTA dephosphorylation, providing new insights into the development of potential therapeutic target.

## Results

### KSHV RTA interacts with the host protein PPP2R1A

**Fig 1:**
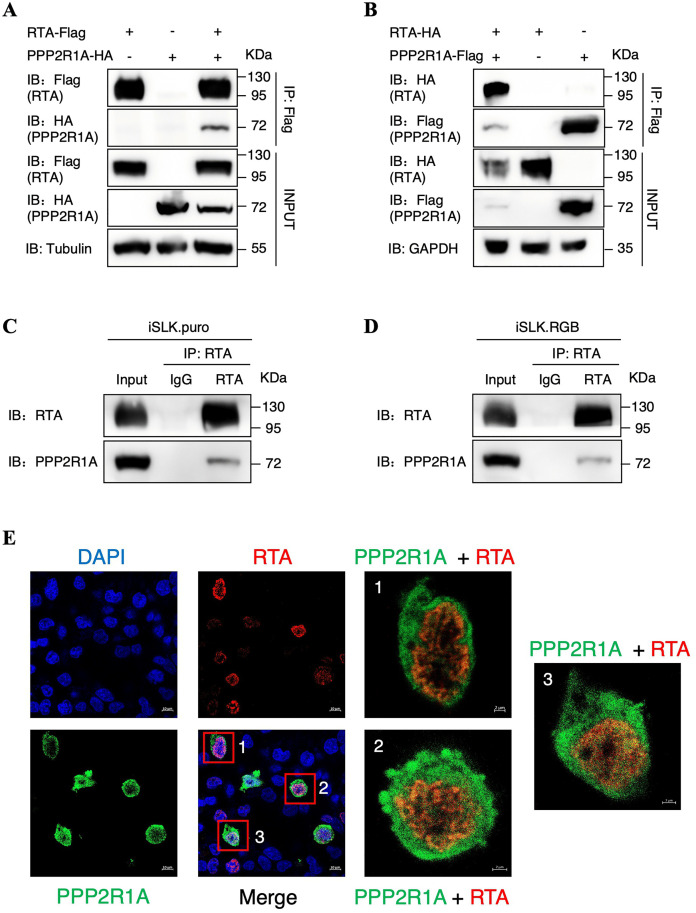
KSHV RTA interacts with the host protein PPP2R1A. **(A)** HEK293T cells were transfected with RTA-Flag alone, with PPP2R1A-HA alone or with both RTA-Flag and PPP2R1A-HA. Cell lysates were immunoprecipitated with an anti-Flag antibody and were then analyzed by western blotting with the indicated antibodies. **(B)** HEK293T cells were transfected with PPP2R1A-Flag alone, with RTA-HA alone or with both PPP2R1A-Flag and RTA-HA. Cell lysates were immunoprecipitated with an anti-Flag antibody and were then analyzed by western blotting with the indicated antibodies. **(C)** Co-IP of endogenous RTA and PPP2R1A in KSHV-negative iSLK.puro cells. Expression of RTA in the cells was induced by doxycycline, and cell lysates were subjected to immunoprecipitation with the anti-RTA antibody or mouse IgG control antibody. Purified proteins, along with input samples, were subjected to western blotting with the indicated antibodies. **(D)** Co-IP of endogenous RTA and PPP2R1A in KSHV-positive iSLK.RGB cells. Lytic replication of KSHV in the cells was induced by doxycycline, and cell lysates were subjected to immunoprecipitation with the anti-RTA antibody or mouse IgG control antibody. Purified proteins, along with input samples, were subjected to western blotting with the indicated antibodies. **(E)** Colocalization of PPP2R1A and RTA in HEK293T cells. Following transfection with RTA-Flag and PPP2R1A-HA, cells were fixed with 4% paraformaldehyde and then stained with anti-HA and anti-Flag antibodies. Secondary antibodies conjugated to FITC- or Cy3-conjugated were used to visualize the stained PPP2R1A and RTA proteins, respectively. Nuclei were labelled with DAPI. Cells were analyzed by Zeiss confocal microscopy and representative images with scale bars were shown.

In our previous study, we identified a series of potential interacting proteins of RTA by Flag-tag-based affinity purification with mass spectrometry analysis in HEK293T and HEK293T.219 cell lines. Among these proteins, PPP2R1A, the scaffold protein of phosphatase PP2A, was spotted to be a candidate protein that can potentially interact with RTA [39]. Therefore, we firstly adopted a two-way co-immunoprecipitation (Co-IP) assay to verify the interaction between RTA and PPP2R1A in HEK293T cells (Fig 1A and 1B). Besides, endogenous PPP2R1A was also demonstrated to form a complex with RTA when RTA expression was induced by doxycycline (Dox) in both iSLK.puro and iSLK.RGB cells (Fig 1C and 1D). Moreover, we performed an immunofluorescence assay (IFA) in HEK293T cells and found that RTA and PPP2R1A are co-localized in the nuclear compartment (Fig 1E). Collectively, these results confirm that the host PPP2R1A is a novel RTA-interacting protein, and their interaction is in the nucleus.

### Ectopic expression of PPP2R1A impairs KSHV lytic replication

**Fig 2:**
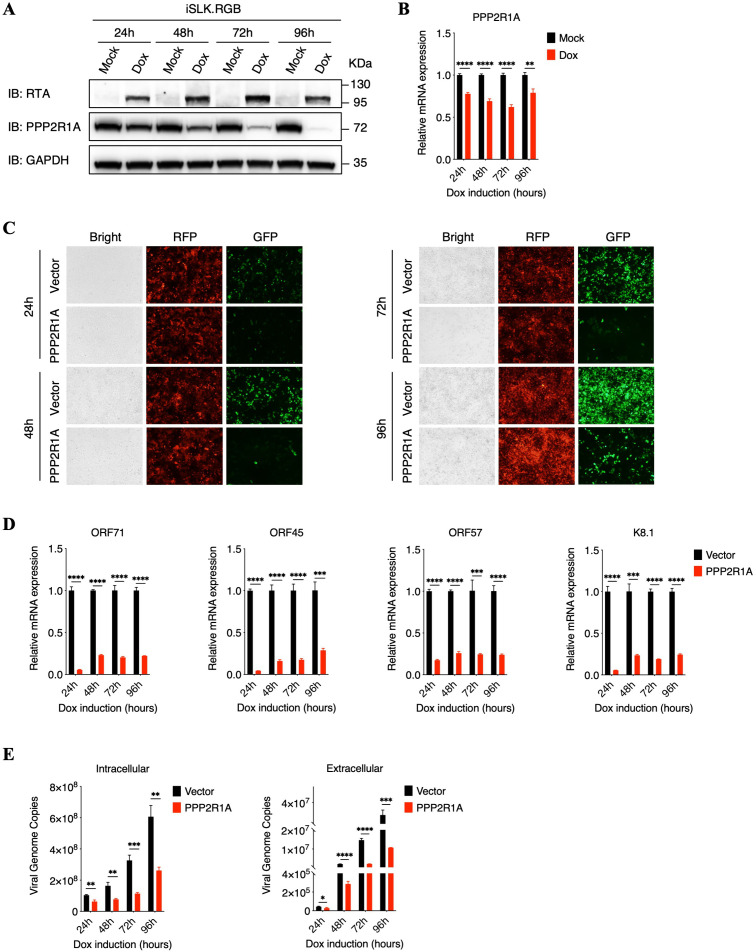
Ectopic expression of PPP2R1A impairs KSHV lytic replication. **(A and B)** iSLK.RGB cells were treated with or without doxycycline. The expression kinetics of RTA and PPP2R1A at indicated time points were detected by immunoblotting (A) and the mRNA expression of PPP2R1A was determined by qPCR analysis (B). **(C)** iSLK.RGB-Vector and iSLK.RGB-PPP2R1A cells were treated with doxycycline at different time points as indicated. Representative bright and fluorescence microscopy images among iSLK.RGB-Vector and iSLK.RGB-PPP2R1A cells were shown. **(D)** PPP2R1A overexpression suppresses the transcription of viral genes. iSLK.RGB-Vector and iSLK.RGB-PPP2R1A cells were treated with doxycycline at different time points as indicated. RNA was extracted from cells to investigate the transcriptional level of several KSHV genes: ORF71, ORF45, ORF57 and K8.1. **(E)** PPP2R1A overexpression suppresses virus production. iSLK.RGB-Vector and iSLK.RGB-PPP2R1A cells were treated with doxycycline at different time points as indicated. Intracellular viral genomic DNA (left panel) and extracellular virion DNA (right panel) were extracted from cell lysates or cell supernatants. Then, the KSHV genomic DNA copy numbers were quantified by qPCR analysis. For B, D and E, bars represent means ±SEM of triplicates from three independent experiments. The P values were calculated using Student’s t-test (two sides). *P < 0.05, **P < 0.01, ***P < 0.001, ****P < 0.0001.

To further determine the role of PPP2R1A in KSHV lytic replication, we firstly detected the kinetics of PPP2R1A expression in iSLK.RGB cells at different time points after doxycycline induction, which demonstrated that both protein and RNA level of PPP2R1A were suppressed, especially at the later time points of viral lytic replication (Fig 2A and 2B). We then constructed an iSLK.RGB-PPP2R1A cell line that stably expressed Flag-tagged PPP2R1A (S1A Fig). Considering that iSLK.RGB cell line contains fluorescent protein expression cassettes, RFP and GFP, which are under the control of the constitutively active EF1α and the immediate early PAN promoters respectively [40], we firstly adopted fluorescence microscopy to evaluate the role of PPP2R1A in KSHV lytic replication, which demonstrated that the number of GFP-positive cells was greatly reduced after PPP2R1A ectopic expression compared with control cells (Fig 2C). Besides, we also demonstrated that the transcripts levels of various genes, including latent and lytic genes of KSHV, were obviously suppressed in PPP2R1A stable expression cell lines (Figs 2D and S1B). Furthermore, comparing with control cells, both extracellular and intracellular KSHV virions were reduced in PPP2R1A overexpressed cells (Fig 2E). Taken together, these results indicated that ectopic expression of PPP2R1A impairs KSHV lytic replication.

### Phosphatase PP2A promotes RTA dephosphorylation

**Fig 3:**
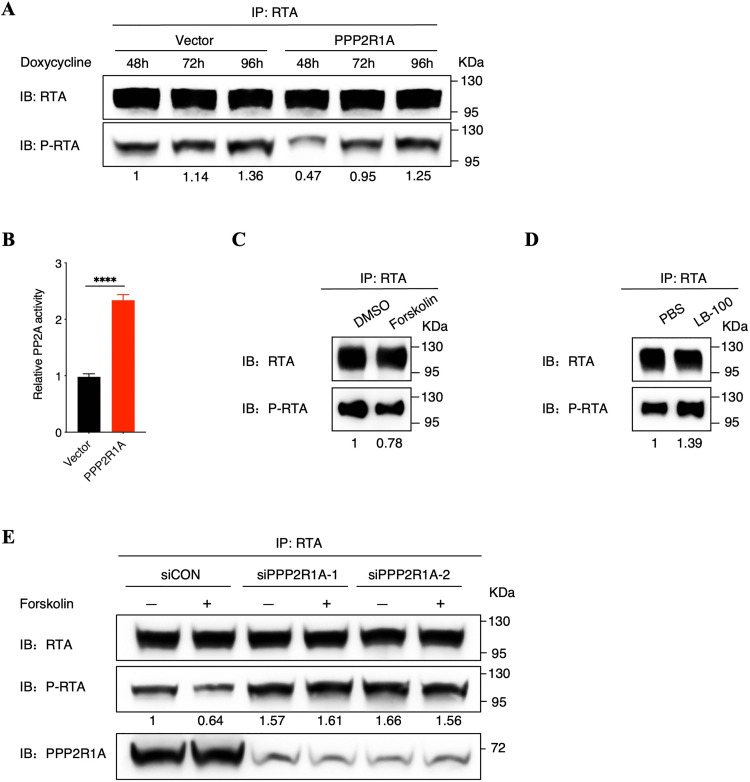
Phosphatase PP2A promotes RTA dephosphorylation. **(A)** PPP2R1A overexpression promotes RTA dephosphorylation. iSLK.RGB-Vector and iSLK.RGB -PPP2R1A cells were induced with doxycycline at different time points as indicated. Cells were then lysed, and cell lysates were immunoprecipitated with an anti-RTA antibody followed by immunoblotting analysis using anti-pan Phospho-Serine/Threonine antibodies. Phosphorylated RTA was quantified by densitometry and normalized to the RTA level. **(B)** PP2A activity was measured among iSLK.RGB-Vector and iSLK.RGB-PPP2R1A cells. Bars represent means ±SEM of triplicates from three independent experiments. The P values were calculated using Student’s t-test (two sides). ****P < 0.0001. **(C)** Phosphatase PP2A agonist Forskolin promotes RTA dephosphorylation. iSLK.RGB cells were induced with doxycycline for 48 hours in the absence and presence of phosphatase PP2A agonist Forskolin (40 μM). WCLs were immunoprecipitated with anti-RTA antibody followed by immunoblotting analysis using anti-pan Phospho-Serine/Threonine antibodies. Phosphorylated RTA was quantified by densitometry and normalized to the RTA level. **(D)** Phosphatase PP2A inhibitor LB-100 enhances RTA phosphorylation. iSLK.RGB cells were induced with doxycycline for 48 hours in the absence and presence of phosphatase PP2A inhibitor LB-100 (5 μM). WCLs were immunoprecipitated with anti-RTA antibody followed by immunoblotting analysis using anti-pan Phospho-Serine/Threonine antibodies. Phosphorylated RTA was quantified by densitometry and normalized to the RTA level. **(E)** Phosphatase PP2A agonist Forskolin cannot promote RTA dephosphorylation when PPP2R1A expression was suppressed with siRNAs. iSLK.RGB cells were transfected with siRNA as indicated. At 24 hours after transfection, cells were induced by doxycycline for 48 hours in the absence and presence of phosphatase PP2A agonist Forskolin (40 μM). WCLs were immunoprecipitated with anti-RTA antibody followed by immunoblotting analysis using anti-pan Phospho-Serine/Threonine antibodies. Phosphorylated RTA was quantified by densitometry and normalized to the RTA level.

Considering PPP2R1A is a scaffold protein of host phosphatase PP2A, which functions as the predominant serine/threonine phosphatase, suggesting phosphatase PP2A potentially regulates the phosphorylation of RTA. To verify this hypothesis, we firstly examined a kinetic analysis of RTA phosphorylation status at different time points during lytic replication in both iSLK.RGB-Vector and iSLK.RGB-PPP2R1A cells. We demonstrated that the phosphorylation of RTA at the different time points show less signals in iSLK.RGB-PPP2R1A cells than that in iSLK.RGB-Vector cells (Fig 3A). Interestingly, in both iSLK.RGB-Vector and iSLK.RGB-PPP2R1A cells, there is a consecutive increase of RTA phosphorylation as lytic replication goes on, probably due to the decrease expression of PPP2R1A. Subsequently, utilizing enzymatic activity experiment, we demonstrated that PP2A activity was greatly enhanced in PPP2R1A stably expressed cells, suggesting that PP2A might have impacts on the phosphorylation of RTA (Fig 3B). We further evaluated RTA phosphorylation after utilizing PP2A agonist (Forskolin) and inhibitor (LB-100) [41,42], which demonstrated that agonist Forskolin effectively reduced the phosphorylation of RTA, while inhibitor LB-100 enhanced the phosphorylation of RTA conversely (Fig 3C and 3D). Of note, when PPP2R1A expression was suppressed with siRNA, RTA phosphorylation was not affected, even if utilizing agonist Forskolin (Fig 3E). Moreover, to further confirm PP2A is responsible for RTA dephosphorylation, we also detected the status of RTA phosphorylation in HEK293T cells co-transfected with RTA and PPP2R1A. Similarly, PPP2R1A overexpression decreases the phosphorylation of RTA in HEK293T cells (S2A Fig). Meanwhile, PP2A agonist Forskolin exacerbates the dephosphorylation of RTA induced by PPP2R1A overexpression, while PP2A inhibitor LB-100 largely counteracts the effect of PPP2R1A on RTA dephosphorylation (S2B and S2C Fig). These results collectively indicated that phosphatase PP2A is responsible for RTA dephosphorylation, which was dependent on the interaction between the scaffold protein PPP2R1A and RTA.

### RTA dephosphorylation mediated by phosphatase PP2A impairs KSHV lytic replication

**Fig 4:**
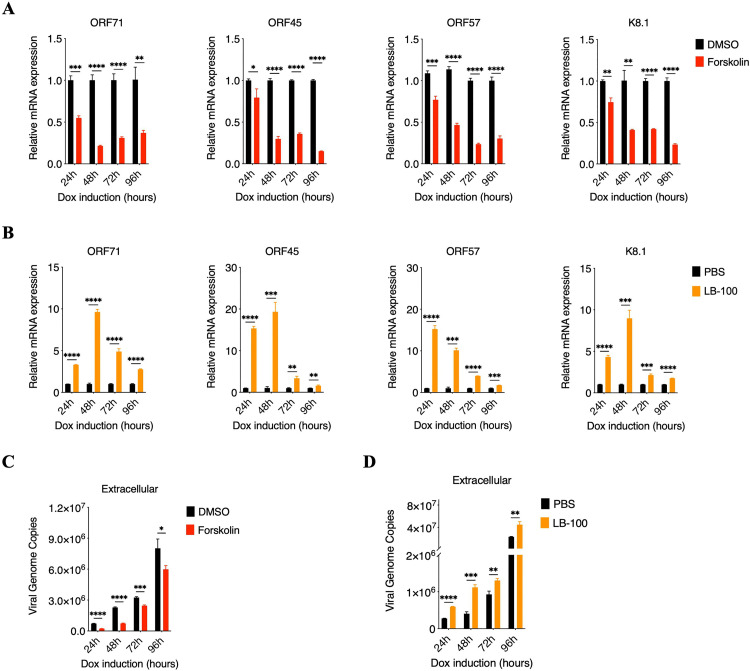
RTA dephosphorylation mediated by phosphatase PP2A impairs KSHV lytic replication. **(A)** Phosphatase PP2A agonist Forskolin suppresses the transcription of viral genes. iSLK.RGB cells were induced with doxycycline at different time points as indicated in the absence and presence of phosphatase PP2A agonist Forskolin (40 μM). RNA was extracted from cells to investigate the transcriptional level of several KSHV genes: ORF71, ORF45, ORF57 and K8.1. **(B)** Phosphatase PP2A inhibitor LB-100 promotes the transcription of viral genes. iSLK.RGB cells were induced with doxycycline at different time points as indicated in the absence and presence of phosphatase PP2A inhibitor LB-100 (5 μM). RNA was extracted from cells to investigate the transcriptional level of several KSHV genes: ORF71, ORF45, ORF57 and K8.1. **(C)** Phosphatase PP2A agonist Forskolin suppresses virus production. iSLK.RGB cells were induced with doxycycline at different time points as indicated in the absence and presence of phosphatase PP2A agonist Forskolin (40 μM). Extracellular virion DNA were extracted from cell supernatants. Then, the KSHV genomic DNA copy numbers were quantified by qPCR analysis. **(D)** Phosphatase PP2A inhibitor LB-100 promotes virus production. iSLK.RGB cells were induced with doxycycline at different time points as indicated in the absence and presence of phosphatase PP2A inhibitor LB-100 (5 μM). Extracellular virion DNA were extracted from cell supernatants. Then, the KSHV genomic DNA copy numbers were quantified by qPCR analysis. For A to D, bars represent means ±SEM of triplicates from three independent experiments. The P values were calculated using Student’s t-test (two sides). *P < 0.05, **P < 0.01, ***P < 0.001, ****P < 0.0001.

Considering the importance of RTA in KSHV lytic replication, we further evaluate the physiological functions of RTA dephosphorylation mediated by phosphatase PP2A in KSHV lytic replication after utilizing agonist Forskolin or inhibitor LB-100. We firstly adopted real-time qPCR to detect viral gene expression, including latent and lytic genes of KSHV, which demonstrated that in multiple time points after doxycycline induction, phosphatase PP2A agonist Forskolin greatly suppressed KSHV gene expression, while phosphatase PP2A inhibitor LB-100 significantly enhanced viral gene expression (Figs 4A, 4B and S3A, S3B). Moreover, we also utilized real-time qPCR to evaluate the production of KSHV progeny virions. Consistent with gene expression, phosphatase PP2A agonist Forskolin impaired the production of KSHV progeny virions (Fig 4C). Conversely, phosphatase PP2A inhibitor promoted the production of KSHV progeny virions (Fig 4D). Taken together, these results indicated that RTA dephosphorylation mediated by phosphatase PP2A impairs KSHV lytic replication, mainly in decreased viral gene expression and the reduction of progeny virions production.

### Phosphatase PP2A promotes RTA dephosphorylation at Thr42 and Thr678 to suppresses the transactivation activity of RTA

**Fig 5:**
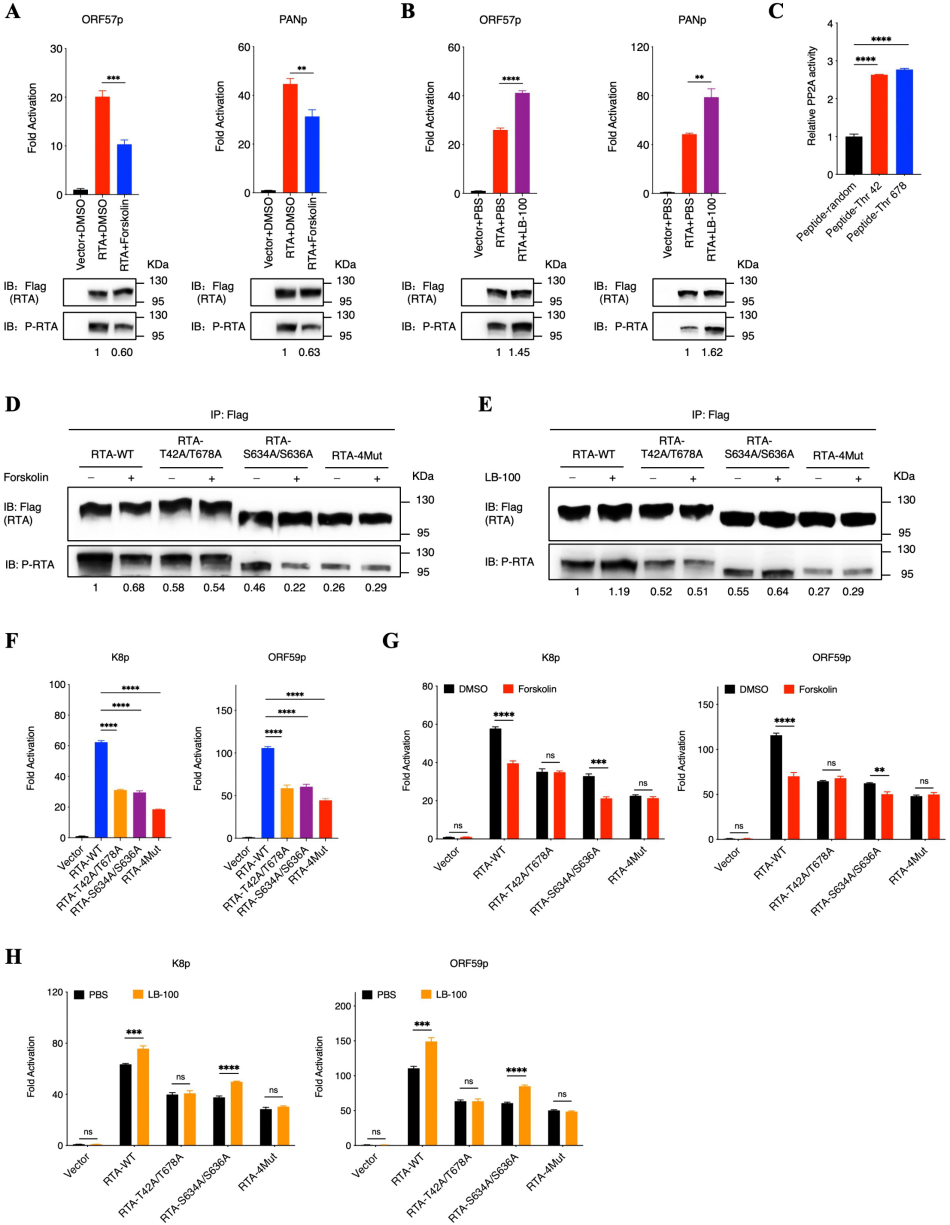
Phosphatase PP2A promotes RTA dephosphorylation at Thr42 and Thr678 to suppresses the transactivation activity of RTA. **(A)** Phosphatase PP2A agonist Forskolin suppresses the transcription activity of RTA. **(B)** Phosphatase PP2A inhibitor LB-100 promotes the transcription activity of RTA. For A and B, HEK293T cells were transfected with ORF57 (left) or PAN (right) reporter plasmids (1 μg) and expression plasmids containing RTA (1 μg) or empty vector (1 μg) as a control. At 6 hours after transfection, cells were treated with 40 μM PP2A agonist Forskolin (A) or 5 μM PP2A inhibitor LB-100 (B) for 48 hours. Cells were then lysed to detect dual luciferase reporter activity and cell lysates were immunoprecipitated with an anti-Flag antibody followed by immunoblotting analysis using anti-pan Phospho-Serine/Threonine antibodies. Phosphorylated RTA was quantified by densitometry and normalized to the RTA level. **(C)** PP2A enzymatic activity was measured in two peptides of RTA containing phosphorylated Thr-42 or Thr-678 sites respectively and one random peptide. **(D and E)** Phosphatase PP2A agonist Forskolin (D) or inhibitor LB-100 (E) has no effect on the phosphorylation status of RTA mutants containing T42A and T678A sites. For D and E, HEK293T cells were transfected with wildtype RTA or RTA mutants as indicated. At 6 hours after transfection, cells were treated with 40 μM PP2A agonist Forskolin (D) or 5 μM PP2A inhibitor LB-100 (E) for 48 hours. Cell lysates were immunoprecipitated with anti-Flag antibody followed by immunoblotting analysis using anti-pan Phospho-Serine/Threonine antibodies. Phosphorylated RTA was quantified by densitometry and normalized to the RTA level. **(F)** The transcriptional activity of three RTA mutants was impaired. HEK293T cells were transfected with K8 (left) or ORF59 (right) reporter plasmids (1 μg) and expression plasmids containing wildtype RTA or RTA mutants as indicated (1 μg) or empty vector (1 μg) as a control. At 48 hours after transfection, cells were then lysed to detect dual luciferase reporter activity. **(G and H)** Phosphatase PP2A agonist Forskolin (G) or inhibitor LB-100 (H) has no effect on the transcriptional activity of RTA mutants containing T42A and T678A sites. For G and H, HEK293T cells were transfected with K8 (left panel) or ORF59 (right) reporter plasmids (1 μg) and expression plasmids containing wildtype RTA or RTA mutants as indicated (1 μg) or empty vector (1 μg) as a control. At 6 hours after transfection, cells were treated with 40 μM PP2A agonist Forskolin (G) or 5 μM PP2A inhibitor LB-100 (H) for 48 hours. Cells were then lysed to detect dual luciferase reporter activity. For A to C and F to H, bars represent means ±SEM of triplicates from three independent experiments. The P values were calculated using Student’s t-test (two sides). **P < 0.01, ***P < 0.001, ****P < 0.0001, ns indicates not significant.

Considering RTA mainly works as the transcriptional factor in KSHV lytic replication to initiate viral gene expression, we next detected whether RTA dephosphorylation mediated by phosphatase PP2A has impacts on the transcriptional activity of RTA. We transfected HEK293T cells with RTA and KSHV lytic promoter plasmids to perform luciferase reporter assays after utilizing phosphatase PP2A agonist or inhibitor. These lytic promoter plasmids contain a series of viral lytic genes like PAN, K8, ORF57, ORF59 and K2. We demonstrated that PP2A agonist Forskolin effectively impaired the transcriptional activity of RTA, while PP2A inhibitor LB-100 conversely enhanced the transcriptional activity of RTA (Figs 5A, 5B and S4A, S4B). These results indicated that RTA dephosphorylation mediated by phosphatase PP2A has impacts on its transcriptional activity.

Subsequently, based on the analysis results of Human Protein Reference Database, we identified two potential phosphorylation sites of RTA, including Thr-42 and Thr-678, which can be recognized by phosphatase PP2A and therefore promoting these two sites of dephosphorylation (S4C Fig). To further confirm the relationship between these two sites and phosphatase PP2A, we firstly synthesized two peptides of RTA containing phosphorylated Thr-42 or Thr-678 respectively and performed enzymatic activity experiment, which demonstrated that the activity of phosphatase PP2A was obviously enhanced in peptides with Thr-42 or Thr-678, compared to the random peptide (Figs 5C and S4D). Next, considering *Tsai* has confirmed that Ser-634 and Ser-636 of RTA are phosphorylated by host transcriptional kinase CDK9, contributing to a full transcriptional function of RTA [23], we thereby constructed three RTA mutants, including two double-sites mutants (T42A/T678A and S634A/S636A) and one quadruple-sites mutant (T42A/S634A/S636A/T678A, 4Mut). We demonstrated that both PP2A agonist Forskolin and PP2A inhibitor LB-100 have obvious impacts on the phosphorylation of both RTA-WT and RTA-S634A/S636A but have little effect on RTA mutants containing T42A and T678A, indicating that phosphatase PP2A promoted RTA dephosphorylation mainly at Thr-42 or Thr-678 sites (Fig 5D and 5E).

Moreover, to further confirm phosphatase PP2A impaired the transcriptional activity of RTA mainly through promoting RTA dephosphorylation at Thr-42 and Thr-678, we firstly evaluated that the transcriptional activity of three RTA mutants, which showed that comparing with wildtype RTA, three RTA mutants all were deprived of capacity in transcriptional activation of various promoters, indicating that the phosphorylation status of RTA is necessary for its transcriptional activity (Figs 5F and S4E). Next, we further analyzed the transcriptional activity of wildtype RTA and three RTA mutants after treating HEK293T cells with PP2A agonist Forskolin or inhibitor LB-100 through luciferase reporter assays. As expected, the transcriptional activity of both RTA-WT and RTA-S634A/S636A were impaired or enhanced in response to the treatment with PP2A agonist or inhibitor respectively, while the transcriptional activity of RTA-T42A/T678A and RTA-4Mut were totally not affected (Fig 5G and 5H). Collectively, these results indicated that phosphatase PP2A promotes RTA dephosphorylation at Thr42 and Thr678, suppressing the transcriptional activity of RTA.

### RTA promotes PPP2R1A degradation through the ubiquitin-proteasome pathway during KSHV lytic replication

**Fig 6:**
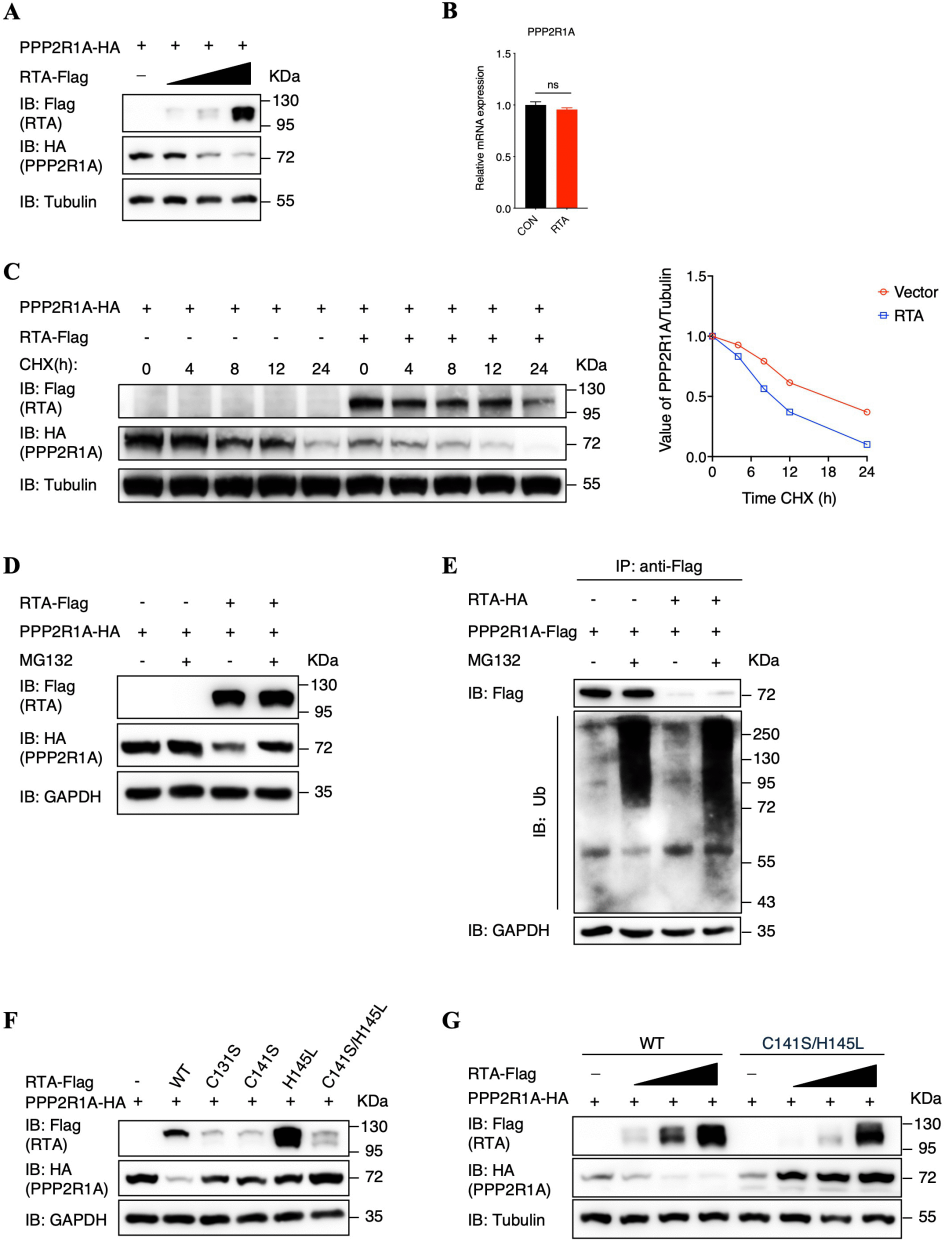
RTA promotes PPP2R1A degradation through the ubiquitin-proteasome pathway during KSHV lytic replication. **(A)** Effect of RTA on PPP2R1A protein expression. HEK293T cells were co-transfected with 1 μg of PPP2R1A expression plasmid and increasing amounts of RTA expression vector (0, 0.5, 1, and 2 μg). At 48 hours after transfection, WCLs were harvested and analyzed by immunoblotting with the indicated antibodies. **(B)** Effect of RTA on endogenous PPP2R1A mRNA level. HEK293T cells were transfected with 2 μg of RTA expression plasmid or vector. At 48 hours after transfection, RNA was collected for reverse transcription and used to quantify PPP2R1A and GAPDH by qPCR analysis. Bars represent means ±SEM of triplicates from three independent experiments. The P values were calculated using Student’s t-test (two sides). ns indicates not significant. **(C)** Measurement of PPP2R1A stability in the absence and presence of RTA. HEK293T cells were transfected with HA-tagged PPP2R1A with or without Flag-tagged RTA expression plasmid for 36 h**.** Cells were treated with 100 μg/ml of CHX and analyzed at indicated time points by immunoblotting for PPP2R1A. Tubulin was used as a control for equivalent sample loading. The relative levels of PPP2R1A were quantified by densitometry and normalized to the Tubulin level. **(D)** RTA promotes the degradation of PPP2R1A through the ubiquitin-proteasome pathway. HEK293T cells were co-transfected with the indicated expression plasmids for 36 h and then treated with 10 μM MG132 or DMSO for another 6 h**.** Cells were lysed and used for western blots with the indicated antibodies. **(E)** HEK293T cells were co-transfected with the indicated expression plasmids for 36 h and then treated with 10 μM MG132 or DMSO for another 6 h**.** Cells were lysed and subjected to immunoprecipitation using anti-Flag antibody, followed by immunoblotting with indicated antibodies to analyze precipitated proteins along with input samples. **(F)** Effect of wildtype RTA or mutant RTA that was deprived of E3 ligase activity on PPP2R1A protein expression. HEK293T cells were co-transfected with 1 μg of PPP2R1A expression plasmid and 1 μg of wildtype RTA or mutant RTA expression plasmids. At 48 hours after transfection, WCLs were harvested and analyzed by immunoblotting with the indicated antibodies. **(G)** Effect of wildtype RTA or mutant RTA that was deprived of E3 ligase activity on PPP2R1A protein expression. HEK293T cells were co-transfected with 1 μg of PPP2R1A expression plasmid and increasing amounts of wildtype or mutant RTA expression plasmids (0, 0.5, 1, and 2 μg). At 48 hours after transfection, WCLs were harvested and analyzed by immunoblotting with the indicated antibodies.

We have demonstrated that PPP2R1A interacts with RTA, promoting RTA dephosphorylation mediated by phosphatase PP2A, which impairs the transactivation activity of RTA and therefore suppressed KSHV lytic replication. Interestingly, PPP2R1A expression was suppressed during viral lytic replication (Fig 2A and 2B), we therefore speculated that KSHV has evolved some mechanisms to evade the antiviral function of PPP2R1A. Considering RTA interacts with PPP2R1A, and works as a E3 ubiquitin ligase in KSHV lytic replication [43–45], we purposed that RTA potentially promotes PPP2R1A degradation. To verify this hypothesis, we firstly performed dose-dependent assays in HEK293T cells, which showed that RTA promotes PPP2R1A protein degradation, while has no impact on the transcript levels of PPP2R1A (Fig 6A and 6B). Meanwhile, we determined the effect of RTA on the half-life of PPP2R1A after treatment with cycloheximide (CHX) in HEK293T cells, which showed that the half-life of PPP2R1A were significantly curtailed in cells overexpressing RTA compared to control cells (Fig 6C). To further verify PPP2R1A degradation was promoted by RTA, we detected endogenous PPP2R1A expression in iSLK.RGB and iSLK.puro cells after suppressing RTA expression with siRNA. We demonstrated that the degradation of PPP2R1A protein was largely abolished when RTA expression was inhibited, while PPP2R1A mRNA levels were not affected (S5A to S5D Fig). These results indicated that PPP2R1A protein degradation was mediated by RTA. Next, to further verify RTA promotes PPP2R1A degradation through ubiquitin-proteasome pathway, we utilized proteasome inhibitor MG132 to treat with HEK293T cells, which demonstrated that MG132 greatly rescued PPP2R1A degradation even in the context of ectopic RTA expression (Fig 6D). Meanwhile, these cells were lysed and immunoprecipitated with an anti-Flag antibody before immunoblotting with an anti-ubiquitin antibody. As expected, the polyubiquitination level of PPP2R1A was greatly increased in RTA overexpressing cells compared with control cells after treatment with MG132 (Fig 6E, lanes 2 and 4). Furthermore, we adopted site-directed mutagenesis to construct four RTA mutants that were deprived of E3 ligase activity, including C131S, C141S, H145L and mutant with C141S and H145L together [43,44]. We demonstrated that these four RTA mutants all failed to promote PPP2R1A degradation (Fig 6F and 6G). In conclusions, these results suggested that RTA promotes PPP2R1A degradation through ubiquitin-proteasome pathway, which antagonizes the anti-viral activity of PPP2R1A.

## Discussion

**Fig 7:**
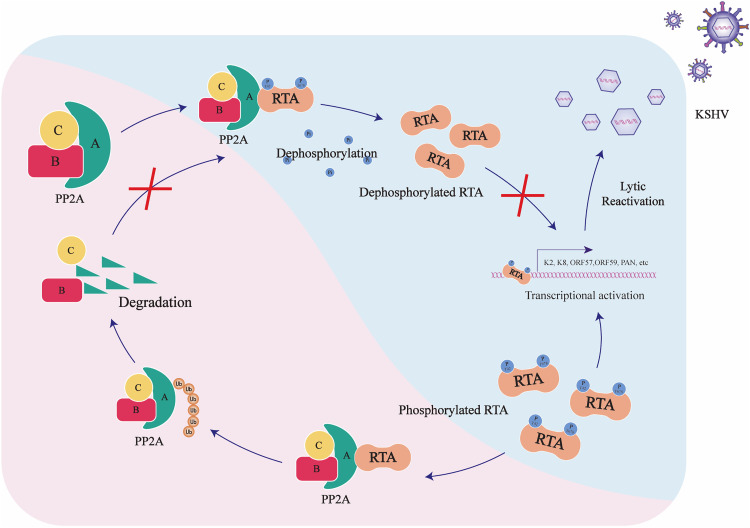
Working model for the role of PPP2R1A in regulating the stability of RTA. The scaffold protein PPP2R1A interacted with RTA, inducing RTA dephosphorylation mediated by phosphatase PP2A, which seriously destroyed the transcription activity of RTA and thereby greatly inhibiting KSHV lytic replication. In turn, RTA promoted PPP2R1A degradation through ubiquitin-proteasome pathway, counteracting the antiviral activity of phosphatase PP2A and ensuring a complete lytic replication of KSHV.

In this study, we found that the scaffold protein PPP2R1A interacts with RTA, inducing RTA dephosphorylation mediated by phosphatase PP2A, which greatly impairs KSHV lytic replication. Mechanistically, utilizing phosphatase PP2A agonist Forskolin and inhibitor LB-100, we demonstrated that RTA dephosphorylation mediated by phosphatase PP2A seriously destroyed the transcription activity of RTA. Meanwhile, we identified two dephosphorylated sites that mediated by phosphatase PP2A and verified their significance in transactivation activity of RTA. Besides, to evade the anti-viral activity of PPP2R1A, KSHV utilized RTA to promote PPP2R1A degradation through ubiquitin-proteasome pathway, ensuring a complete lytic replication (Fig 7).

Phosphatase PP2A works as the major serine/threonine phosphatase in the cell and therefore participates in many cellular processes, including regulation of signal transduction pathways, cell cycle progression, DNA replication, gene transcription, and protein translation [25,31,34,36,38]. However, the function and mechanism of PP2A in KSHV life cycle remain elusive, only Shamay and colleague, in an early study, have reported that the ability of LANA to interact with the B subunit of PP2A disrupts the formation of PP2A holoenzyme, but this interaction has no effect on LANA expression [46], suggesting that PP2A potentially takes part in KSHV lifecycle through promoting dephosphorylation of other cellular and host target proteins. Our studies identified one of the novel RTA-interacting proteins, the A subunit of phosphatase PP2A, and confirmed that PP2A promotes the dephosphorylation of RTA through interaction between A subunit of PP2A and RTA, which complemented the understanding of the functions of PP2A and underscored the biological significance of PP2A in KSHV lytic reactivation.

Moreover, RTA is the key lytic switch protein, controlling reactivation from latency and the induction of lytic replication [21,47,48]. Although RTA is highly phosphorylated during viral replication [12], only *Tsai* colleagues has confirmed that Ser-634 and Ser-636 of RTA are phosphorylated by host transcriptional kinase CDK9, contributing to a full transcriptional function of RTA [23]. Our studies for the first time identified RTA phosphatase PP2A and confirmed additional two novel phosphorylated sites of RTA, Thr-42 and Thr-678, that were recognized by phosphatase PP2A. These results collectively complement the reversible cycle of RTA phosphorylation, emphasize the significance of RTA phosphorylation in KSHV lytic replication.

In addition to lytic replication, RTA is also expressed in KSHV *de novo* infection and plays an essential role in the regulation of viral primary infection [49–51]. Therefore, we further explored the function and biological significance of interaction between PPP2R1A and RTA in KSHV *de novo* infection. We firstly constructed a cell line that stably expressed Flag-tagged PPP2R1A in SLK cells, named SLK-PPP2R1A (S6A Fig), and utilized the recombinant rKSHV.219 to perform KSHV *de novo* infection experiments. We demonstrated that PPP2R1A stable expression effectively suppressed KSHV lytic genes transcriptional levels, including immediate early (IE), early (E) and late genes (L) (S6B Fig). Besides, we also detected viral genes transcriptional levels after inhibiting PPP2R1A expression with siRNAs in KSHV *de novo* infection. As expected, the transcriptional levels of KSHV lytic genes were obviously increased after suppressing PPP2R1A expression (S6C and S6D Fig). These results collectively showed that PPP2R1A functions as a major repressor of lytic genes during KSHV *de novo* infection, which possibly through promoting the dephosphorylation of RTA. To confirm this possibility, we detected a kinetic analysis of RTA phosphorylation status at different time points during *de novo* infection in both SLK-Vector and SLK-PPP2R1A cells, which demonstrated that the phosphorylation of RTA shows less signals in SLK-PPP2R1A cells than that in SLK-Vector cells (S6E Fig). Moreover, when primary infection goes on, the consecutive decrease of RTA phosphorylation was observed in both SLK-Vector and SLK-PPP2R1A cells, potentially destroyed the functions of RTA and thereby inhibiting lytic genes transcriptional levels. Taken together, we demonstrated that PPP2R1A can promote RTA dephosphorylation in both KSHV *de novo* infection and lytic replication, broadened our understanding of RTA phosphorylation and underscored the complicated and diversity of the interaction between RTA and PPP2R1A in KSHV lifecycle.

Additionally, it has been reported that the sequence and function of RTA is highly conserved among the rhadinoviruses [52], we speculated that the dephosphorylation of RTA mediated by phosphatase PP2A is common among gamma herpesviruses. We therefore selected another kind of RTA, MHV68 RTA, in rhadinoviruses for further detection. Firstly, we detected the phosphorylation of MHV68 RTA after utilizing phosphatase PP2A agonist or inhibitor. Consistent with KSHV, the phosphorylation of MHV68 RTA was obviously enhanced when treating cells with phosphatase PP2A inhibitor LB-100, while phosphatase PP2A agonist Forskolin greatly suppressed the phosphorylation of RTA in MHV68 conversely (S7A and S7B Fig). Besides, considering RTA shares a common function in transcriptional activation [53], we constructed corresponding promoter plasmid for MHV68 and performed luciferase reporter assays after utilizing phosphatase PP2A agonist or inhibitor. Similarly, phosphatase PP2A agonist Forskolin effectively suppressed the transactivation activity of MHV68 RTA, while phosphatase PP2A inhibitor LB-100 obviously enhanced its functions (S7C and S7D Fig). These results collectively indicated that phosphatase PP2A-induced the dephosphorylation of RTA is conserved among rhadinoviruses, undermining the transactivation activity of RTA.

Moreover, PP2A is a well-known serine/threonine phosphatase that manipulate diverse cellular signaling pathways through dephosphorylation, which plays a critical role in inflammation, Alzheimer’s disease and various cancer progresses, including tumor initiation, progression, prognosis, and treatment [54]. However, little research about the functions of PP2A in viral lifecycle. Our studies firstly confirmed the antiviral activity of phosphatase PP2A in herpesviruses, shed light on an additional function of PP2A. Considering phosphorylation have significant impacts on various viral proteins, including SARS-CoV-2 nucleocapsid protein [55,56], influenza A virus NS1 and PA proteins [57,58], HIV-1 envelope glycoprotein 120 (gp120) [59], and HSV-1 US3 and UL13 proteins [60,61], etc., regulating their localization, stability, enzymatic activity and functions, which suggests phosphatases might participate in various viral infection, broadening our understanding of phosphatases and providing a potential therapeutic target for the treatment of diseases induced by viral infection.

## Materials and methods

### Cell culture

The HEK293T, iSLK.puro, iSLK.RGB and SLK cell lines were maintained in high-glucose Dulbecco’s modified Eagle’s medium (DMEM, Biological Industries), supplemented with 10% fetal bovine serum (FBS, Biological Industries), 1% antibiotics (penicillin and streptomycin, Gibco) and proper selective agents (puromycin, 1.5 μg/ml; G418, 0.5 mg/ml; hygromycin, 0.5 mg/ml). All cells were cultured at 37˚C in a humidified environment supplemented with 5% CO_2_.

### Antibodies and reagents

The following primary antibodies were used: anti-PPP2R1A rabbit monoclonal antibody (Abcam, ab24736), anti-UBC rabbit polyclonal antibody (Abclonal, A3207), anti-Rabbit Control IgG antibody (Abclonal, AC005), anti-GAPDH mouse monoclonal antibody (Abclonal, AC033), anti-β-actin rabbit monoclonal antibody (Abclonal, AC026), anti-α-tubulin mouse monoclonal antibody (Sigma, T6199), anti-Flag antibody (Sigma, F7425 and F1804), anti-HA antibody (Sigma, H9658 and H6908) and anti-RTA rabbit and mouse polyclonal antibody were prepared in our laboratory. The secondary antibodies used in western blotting and immunofluorescence assays were HRP-conjugated anti-mouse or anti-rabbit IgG (Jackson ImmunoResearch Laboratories) and goat anti-mouse antibodies conjugated with Alexa Fluor 488 (Thermo Fisher Scientific, A-11029) and 555 (Thermo Fisher Scientific, A-21422). The other used reagents and their sources were as follows: recombinant protein A agarose (Invitrogen, 15948–014), recombinant protein G agarose (Invitrogen, 15920–010), anti-Flag M2 affinity gel (Sigma, A2220), Lipofectamine 2000 (ThermoFisher Scientific, 11668019), MG132 (MedChemExpress, HY-13259), cycloheximide (CHX) (MedChemExpress, HY-12320), protease inhibitor cocktail (Sigma, P8340), phosphatase PP2A inhibitor (LB-100, MedChemExpress, HY-18597) and phosphatase PP2A agonist (Forskolin, MedChemExpress, HY-15371).

### Plasmids

The full-length fragment of PPP2R1A and RTA were amplified from a iSLK.RGB cDNA library, inserted into streptomycin-flag-tagged pCDH and HA-tagged pCMV vectors. The RTA mutant plasmids were generated by using the pCMV-HA-RTA plasmid as a template following the manufacturer’s protocol of the Fast Site-Directed Mutagenesis Kit (TIANGEN). Flag-tagged MHV68-RTA and pMHV68-RTA plasmids was a gift from Dr. Xiaozhen Liang’s lab (Shanghai Institute of Immunity and Infection, Chinese Academy of Sciences). The luciferase reporter plasmids pGL3-Basic-pK8, pORF59, pPAN, pORF57 and pK2 were constructed by cloning the promoter regions of respective genes (-2000 to -1 bp) from the iSLK.RGB genomic library into the pGL3-Basic vector. The PCR primers used in this study were summarized in S1 Table.

### KSHV and *de novo* KSHV infection

rKSHV.219 was prepared from iSLK.219 cell cultures by treating the cells with doxycycline (1 μg/ml) and in the absence of G418, hygromycin and puromycin. Four or five days later, the supernatant was collected and cleared of cells and debris by centrifugation (1500 x g for 15 mins at 4°C), followed by filtration with 0.45-μm-pore-size membrane. *De novo* infection of SLK cells were achieved by centrifugation (2500 x rpm for 120 mins at 37°C) after the addition of concentrated virus to the medium, and the medium was changed at 2 hours postinfection.

### Coimmunoprecipitation (co-IP) and Immunoblotting

Treated cells were lysed in RIPA buffer (Beyotime, P0013D) supplemented with protease inhibitor cocktail and 1mM PMSF on Rotational Incubator for 1 h at 4˚C. Then the cells were centrifuged at 13,000 rpm for 15 min at 4˚C to remove cell debris. Five to ten percent of the cell lysates were taken as the input, and the remainder was immunoprecipitated with affinity beads or the corresponding antibodies overnight at 4˚C. The immunoprecipitated were washed three times with RIPA buffer and boiled in SDS loading buffer at 100˚C for 10 minutes. For immunoblotting analysis, the treated protein samples were analyzed by SDS-PAGE and transferred to nitrocellulose membranes, followed by blocking with 5% skim milk powder in TBST buffer for 1 h at room temperature and probing with the indicated primary antibodies overnight at 4˚C. After hybridization with either goat anti-rabbit or goat anti-mouse secondary antibodies (diluted 1:5000) in TBST buffer, membranes were washed with TBST buffer three times (5–10 mins each) before visualization with ECL reagents (GE).

### Immunofluorescence assay

HEK293T cells were plated onto coverslips in 12-well plates. At 48 h prior to transfection, cells were washed thrice with PBS and fixed in 4% paraformaldehyde for 30 mins. Cells were permeabilized with 0.2% Triton X-100 for 15 min and blocked for 30 min with 5% bovine serum albumin (BSA) in PBS, followed by incubation with the primary antibody overnight at 4˚C. After thrice washes with PBS, cells were incubated with FITC- or Cy3-conjugated secondary antibodies for 1 h at room temperature. Cell nuclei were stained with DAPI (Beyotime, C1002) for 5 mins. Finally, the coverslips were washed extensively and fixed onto slides. Slides were visualized and photographed by Zeiss confocal microscopy.

### RNA isolation and quantitative real-time (RT-qPCR)

Total RNA was isolated from cells using TRIzol reagent (Invitrogen) following the manufacturer’s instructions. Two micrograms of RNA was used for reverse transcription with gDNA Eraser reverse transcription kits (Toyobo). cDNA was used for quantification of the indicated mRNA on a QuantStudio 6 Flex Real-Time PCR System (Applied Biosystems) by using SYBR Green real-time PCR master mix kits (Toyobo) according to the manufacturer’s instructions. Dissociation curve analysis of products was conducted at the end of each PCR to detect and validate the specific amplification of PCR products. Transcript levels of each gene were normalized to the GAPDH level, and the 2 − ΔΔCT method was used to analyze gene expression in samples. To analyze viral genomic DNA level, intracellular viral genomic DNA and extracellular virion DNA were extracted from induced cells or the cell supernatants with the Genomic DNA Extraction Kit (TIANGEN). The KSHV genomic DNA copy numbers were quantified by RT-qPCR using primers K9. The standard curve was generated using serial dilutions of a PCDNA3.1-K9 plasmid. All the samples were tested in triplicate. The primers used in RT-qPCR were listed in S1 Table.

### Cycloheximide chase assay

HEK293T cells were co-transfected with the indicated plasmids. At 24 h post-transfection, cells were treated with 100 μg/ml of CHX to inhibit *de novo* protein synthesis. At the indicated time points after treatment with CHX, the cells were harvested and subjected to immunoblot analysis. The intensity of each PPP2R1A band was first normalized with the intensity of its corresponding GAPDH, followed by comparing to the normalized PPP2R1A value at 0 h, and data was analyzed by GraphPad Prism software.

### Ubiquitination assay

HEK293T cells were co-transfected with indicated plasmids and then treated with MG132 (20 μM) or DMSO for 12 h. After 48 h post-transfection, the cells were lysed and immunoprecipitated with anti-Flag antibody, followed by the precipitates were analyzed by immunoblotting using anti-UBC rabbit polyclonal antibody.

### Dual-luciferase reporter assay

HEK293T cells were cultured in 12-well plates followed by transfecting with the indicated expression plasmids. At 24 or 48 h post-transfection, cells were washed twice with 1 × PBS and lysed with 200 μl 1 × passive lysis buffer for 30 mins at room temperature. 10 μl cell lysates were used to measure luminescence activity according to the manufacture’s instruction of dual-luciferase reporter assay system (Promega). The expressing Renilla luciferase plasmids pRL-TK were used to normalize firefly luciferase activity.

### Protein phosphatase activity assay

Phosphatase activity assay was performed using a protein phosphatase assay kit (Promega, USA). Cell lysates or peptides were incubated with phosphor-substrate at room temperature for 30 mins. At the end of the incubation, add stop solution (sodium hydroxide) to stop the reaction. Measure the absorbance of free phosphates at 405 nm and calculated the PP2A activity according to the manufacturer’s instructions.

### RNA interference

iSLK.RGB cells were transfected with negative control siRNA or siRNAs corresponding to the indicated genes (GenePharma Technology) using Lipofectamine 2000 according to the manufacturer’s instructions. At 24 ~ 48 h post-transfection, the cells were harvested, and the efficiency of RNA interference was detected by immunoblotting analysis. All siRNA used in our experiments and their sequences are as follows:

Negative control siRNA, 5’-UUCUCCGAACGUGUCACGUTT-3’

siRTA-1, 5’-GUGCCGUGUAGAGAUUCAATT-3’

siRTA-2, 5’-GUACCUCUUUGGGAUCAAUTT-3’

siPPP2R1A-1, 5’-GGAGUUCUUUGAUGAGAAATT-3’

siPPP2R1A-2, 5’-GACUAGGAGUAGCUUGUUATT-3’

### Establishment and identification of stable cell lines

PPP2R1A-overexpressing lentiviruses were constructed based on the lentiviral vector pCDH-CMV-Flag-IRES-Blast. This PPP2R1A-overexpressing lentiviral vector and empty vectors were packaged in HEK293T cells by co-transfection with the Δ8.9 packaging plasmid and a plasmid expressing vesicular stomatitis virus G protein (pVSV-G). At 48 h post-transfection, the virus stock was collected and cleared by a 0.45 μm pore size filter. The PPP2R1A stably expressing iSLK.RGB and SLK cell lines were achieved by addition of the PPP2R1A stable expression lentiviral particles and centrifugation at 2500 rpm for 2 h. The medium was replaced by fresh DMEM. At 48 h post-infection, the cells were screened with 25 μg/ml blasticidin (Sigma), and immunoblotting was performed to determine the expression of PPP2R1A.

### Statistical analysis

GraphPad Prism software was used to perform the statistical analysis. Data were determined by the unpaired, two tailed Student’s t-tests and statistical significance was set as: ns, no significance, P-value >0.05; *, P-value<0.05; **, P-value<0.01; ***, P-value<0.001; ****, P-value<0.0001. Error bars represent as mean ± SD. Each experiment was carried out independently at least three times.

## Supporting information

S1 Fig Ectopic expression of PPP2R1A impairs KSHV lytic replication. (A) iSLK.RGB cells were stably transduced with lentiviruses containing an empty vector plasmid or a Flag-tagged PPP2R1A expression plasmid and were named as iSLK.RGB-Vector or iSLK.RGB-PPP2R1A cells, respectively. Overexpression of PPP2R1A was detected by western blotting. (B) PPP2R1A overexpression suppresses the transcription of viral genes. iSLK.RGB-Vector and iSLK.RGB-PPP2R1A cells were treated with doxycycline at different time points as indicated. RNA was extracted from cells to investigate the transcriptional level of several KSHV genes: ORF73, ORF59, ORF65 and ORF48. For B, bars represent means ±SEM of triplicates from three independent experiments. The P values were calculated using Student’s t-test (two sides). **P < 0.01, ****P < 0.0001.
(TIF)

S2 Fig Phosphatase PP2A promotes RTA dephosphorylation. (A) PPP2R1A overexpression promotes RTA dephosphorylation. (B) Phosphatase PP2A agonist Forskolin exacerbates the effect of PPP2R1A on RTA dephosphorylation. (C) Phosphatase PP2A inhibitor LB-100 counteracts the effect of PPP2R1A on RTA dephosphorylation. For A to C, HEK293T cells were transfected with RTA-Flag alone or with both RTA-Flag and PPP2R1A-HA for 48 hours. For B and C, at 6 hours after transfection, cells were treated with 40 μM PP2A agonist Forskolin (B) or 5 μM PP2A inhibitor LB-100 (C). Cell lysates were then immunoprecipitated with an anti-Flag antibody followed by immunoblotting analysis using anti-pan Phospho-Serine/Threonine antibodies. Phosphorylated RTA was quantified by densitometry and normalized to the RTA level.
(TIF)

S3 Fig RTA dephosphorylation mediated by phosphatase PP2A impairs KSHV lytic replication. (A) Phosphatase PP2A agonist Forskolin suppresses the transcription of viral genes. iSLK.RGB cells were induced with doxycycline at different time points as indicated in the absence and presence of phosphatase PP2A agonist Forskolin (40 μM). RNA was extracted from cells to investigate the transcriptional level of several KSHV genes: ORF73, ORF59, ORF65 and ORF48. (B) Phosphatase PP2A inhibitor LB-100 promotes the transcription of viral genes. iSLK.RGB cells were induced with doxycycline at different time points as indicated in the absence and presence of phosphatase PP2A inhibitor LB-100 (5 μM). RNA was extracted from cells to investigate the transcriptional level of several KSHV genes: ORF73, ORF59, ORF65 and ORF48. For A and B, bars represent means ±SEM of triplicates from three independent experiments. The P values were calculated using Student’s t-test (two sides). **P < 0.01, ***P < 0.001, ****P < 0.0001.
(TIF)

S4 Fig Phosphatase PP2A promotes RTA dephosphorylation at Thr42 and Thr678 to suppresses the transactivation activity of RTA. (A) Phosphatase PP2A agonist Forskolin suppresses the transcription activity of RTA. (B) Phosphatase PP2A inhibitor LB-100 promotes the transcription activity of RTA. For A and B, HEK293T cells were transfected with K8 (left), ORF59 (middle) or K2 (right) reporter plasmids (1 μg) and expression plasmids containing RTA (1 μg) or empty vector (1 μg) as a control. At 6 hours after transfection, cells were treated with 40 μM PP2A agonist Forskolin (A) or 5 μM PP2A inhibitor LB-100 (B) for 48 hours. Cells were then lysed to detect dual luciferase reporter activity and cell lysates were immunoprecipitated with an anti-Flag antibody followed by immunoblotting analysis using anti-pan Phospho-Serine/Threonine antibodies. Phosphorylated RTA was quantified by densitometry and normalized to the RTA level. (C) The chart shows the potential dephosphorylation sites of RTA in the Human Protein Reference Database. (D) The chart shows the two peptides of RTA containing phosphorylated Thr-42 or Thr-678 respectively and one random peptide. (E) The transcriptional activity of three RTA mutants was impaired. HEK293T cells were transfected with PAN (left), ORF57 (middle) or K2 (right) reporter plasmids (1 μg) and expression plasmids containing wildtype RTA or RTA mutants as indicated (1 μg) or empty vector (1 μg) as a control. At 48 hours after transfection, cells were then lysed to detect dual luciferase reporter activity. For A, B and E, bars represent means ±SEM of triplicates from three independent experiments. The P values were calculated using Student’s t-test (two sides). **P < 0.01, ***P < 0.001, ****P < 0.0001.
(TIF)

S5 Fig RTA promotes PPP2R1A degradation through the ubiquitin-proteasome pathway during KSHV lytic replication. (A to D) iSLK.RGB (A and B) or iSLK.puro (C and D) cells were transfected with siRNAs as indicated. At 24 hours after transfection, cells were induced with doxycycline at different time points as indicated. The expression kinetics of RTA and PPP2R1A at indicated time points were detected by immunoblotting (A or C) and the mRNA expression of PPP2R1A was determined by qPCR analysis (B or D). For B and D, bars represent means ±SEM of triplicates from three independent experiments. The P values were calculated using Student’s t-test (two sides). ns indicates not significant.
(TIF)

S6 Fig PPP2R1A works as a major repressor of viral lytic genes during KSHV *de novo* infection. (A) SLK cells were stably transduced with lentiviruses containing an empty vector plasmid or a Flag-tagged PPP2R1A expression plasmid and were named as SLK-Vector or SLK-PPP2R1A cells, respectively. Overexpression of PPP2R1A was detected by western blotting. (B) SLK-Vector and SLK-PPP2R1A cells were infected with rKSHV.219 for 48 hours. Cells were then lysed, and RNA were extracted to examine the transcriptional level of several KSHV genes: ORF45, RTA, ORF6, ORF36, ORF25 and ORF64. (C) SLK cells were transfected with siRNAs as indicated. The knockdown efficiency of PPP2R1A was determined by western blotting. (D) SLK cells were transfected with siRNAs targeting PPP2R1A for 24 hours and then infected with rKSHV.219 for 48 hours. Cells were then lysed, and RNA were extracted to examine the transcriptional level of several KSHV genes: ORF45, RTA, ORF6, ORF36, ORF25 and ORF64. (E) SLK-Vector and SLK-PPP2R1A cells were infected with rKSHV.219 at different time points as indicated. Cells were then lysed, and cell lysates were immunoprecipitated with an anti-RTA antibody followed by immunoblotting analysis using anti-pan Phospho-Serine/Threonine antibodies. Phosphorylated RTA was quantified by densitometry and normalized to the RTA level. For B and C, bars represent means ±SEM of triplicates from three independent experiments. The P values were calculated using Student’s t-test (two sides). *P < 0.05, **P < 0.01, ***P < 0.001, ****P < 0.0001.
(TIF)

S7 Fig RTA dephosphorylation mediated by phosphatase PP2A is conserved among rhadinoviruses. (A) Phosphatase PP2A inhibitor LB-100 enhances the phosphorylation of MHV68 RTA. HEK293T cells were transfected with 2 μg MHV68 RTA plasmids. At 24 hours after transfection, cells were treated with LB-100 (5 μM) or PBS for 48 hours. WCLs were immunoprecipitated with anti-Flag antibody followed by immunoblotting analysis using anti-pan Phospho-Serine/Threonine antibodies. Phosphorylated RTA was quantified by densitometry and normalized to the RTA level. (B) Phosphatase PP2A agonist Forskolin promotes the dephosphorylation of MHV68 RTA. HEK293T cells were transfected with 2 μg MHV68 RTA plasmids. At 24 hours after transfection, cells were treated with Forskolin (40 μM) or DMSO for 48 hours. WCLs were immunoprecipitated with anti-Flag antibody followed by immunoblotting analysis using anti-pan Phospho-Serine/Threonine antibodies. Phosphorylated RTA was quantified by densitometry and normalized to the RTA level. (C) Phosphatase PP2A agonist Forskolin suppresses the transcription activity of MHV68 RTA. Dual luciferase assay was performed in HEK293T cells. Cells were transiently transfected with MHV68 RTA reporter plasmids (100 ng) and expression plasmids containing MHV68 RTA (1 μg) or empty vector (1μg) as a control. At 6 hours after transfection, HEK293T cells were treated with Forskolin (40 μM) or DMSO for 48 hours, then cells were lysed, and luciferase activity was detected. (B) Phosphatase PP2A inhibitor LB-100 promotes the transcription activity of MHV68 RTA. Dual luciferase assay was performed in HEK293T cells. Cells were transiently transfected with MHV68 RTA reporter plasmids (100 ng) and expression plasmids containing MHV68 RTA (1 μg) or empty vector (1μg) as a control. At 6 hours after transfection, HEK293T cells were treated with LB-100 (5 μM) or PBS for 48 hours, then cells were lysed, and luciferase activity was detected. For C and D, bars represent means ±SEM of triplicates from three independent experiments. The P values were calculated using Student’s t-test (two sides). ***P < 0.001, ****P < 0.0001.
(TIF)

S1 Table Primers used in this study. (XLSX)

S2 Table Raw data. (XLSX)
