# Supplementary figures and images for: Phosphatase PP2A promotes RTA dephosphorylation to impair KSHV lytic replication
Source: PLoS Pathog. 2025 Dec 3;21(12):e1013731. doi: 10.1371/journal.ppat.1013731 (PMC12674568; doi:10.1371/journal.ppat.1013731)

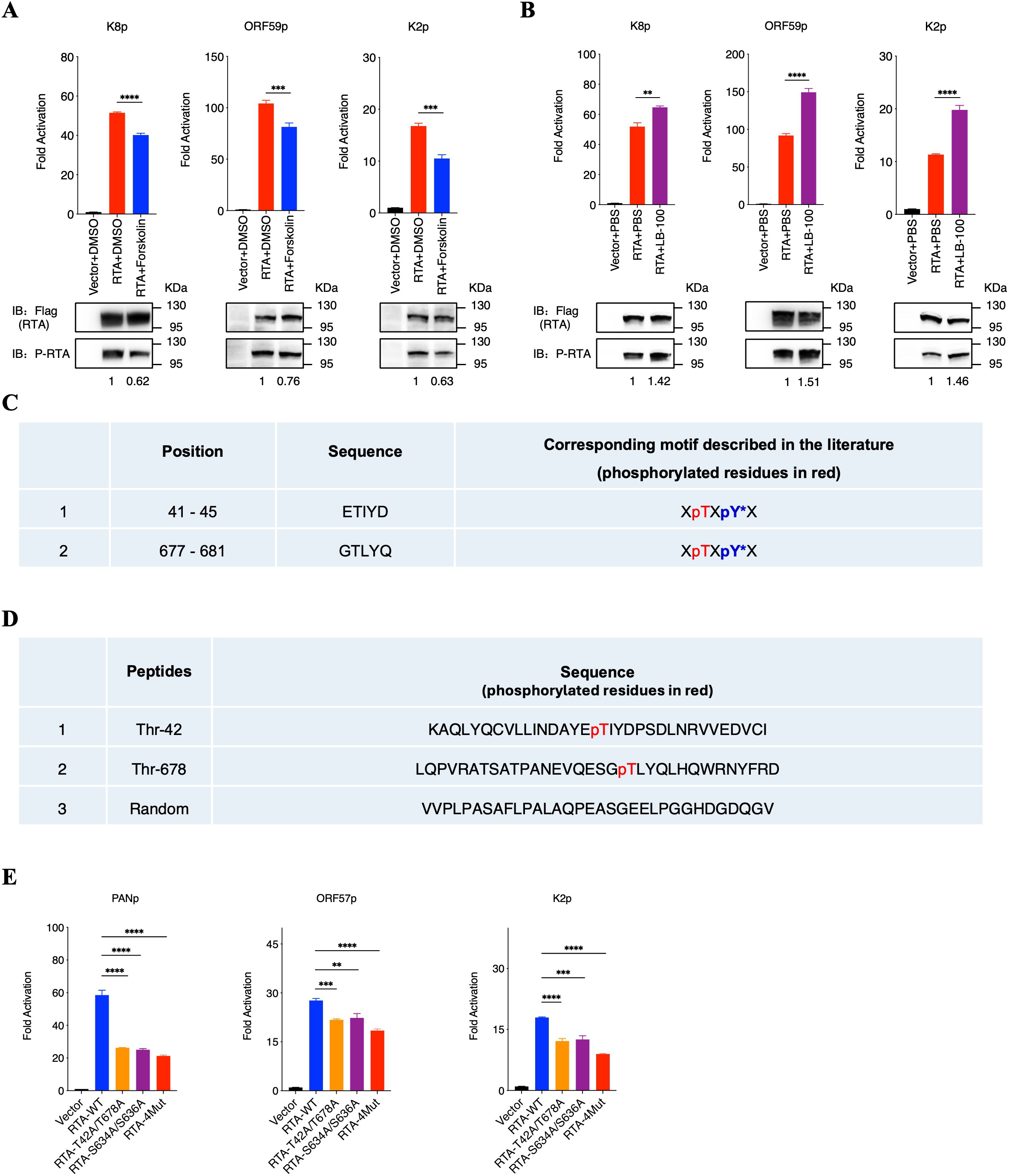

Supplement: S4 Fig — (A) Phosphatase PP2A agonist Forskolin suppresses the transcription activity of RTA. (B) Phosphatase PP2A inhibitor LB-100 promotes the transcription activity of RTA. For A and B, HEK293T cells were transfected with K8 (left), ORF59 (middle) or K2 (right) reporter plasmids (1 μg) and expression plasmids containing RTA (1 μg) or empty vector (1 μg) as a control. At 6 hours after transfection, cells were treated with 40 μM PP2A agonist Forskolin (A) or 5 μM PP2A inhibitor LB-100 (B) for 48 hours. Cells were then lysed to detect dual luciferase reporter activity and cell lysates were immunoprecipitated with an anti-Flag antibody followed by immunoblotting analysis using anti-pan Phospho-Serine/Threonine antibodies. Phosphorylated RTA was quantified by densitometry and normalized to the RTA level. (C) The chart shows the potential dephosphorylation sites of RTA in the Human Protein Reference Database. (D) The chart shows the two peptides of RTA containing phosphorylated Thr-42 or Thr-678 respectively and one random peptide. (E) The transcriptional activity of three RTA mutants was impaired. HEK293T cells were transfected with PAN (left), ORF57 (middle) or K2 (right) reporter plasmids (1 μg) and expression plasmids containing wildtype RTA or RTA mutants as indicated (1 μg) or empty vector (1 μg) as a control. At 48 hours after transfection, cells were then lysed to detect dual luciferase reporter activity. For A, B and E, bars represent means ±SEM of triplicates from three independent experiments. The P values were calculated using Student’s t-test (two sides). **P < 0.01, ***P < 0.001, ****P < 0.0001. (TIF) [file ppat.1013731.s004.tif]
